# Replicative genetic association study between functional polymorphisms in *AVPR1A* and social behavior scales of autism spectrum disorder in the Korean population

**DOI:** 10.1186/s13229-017-0161-9

**Published:** 2017-08-09

**Authors:** So Young Yang, Soon Ae Kim, Gang Min Hur, Mira Park, Jong-Eun Park, Hee Jeong Yoo

**Affiliations:** 1 Department of Pharmacology, School of Medicine, Eulji University, Daejeon, Republic of Korea; 2 Department of Pharmacology, College of Medicine, Chungnam National University, Daejeon, Republic of Korea; 3 Department of Preventive Medicine, School of Medicine, Eulji University, Daejeon, Republic of Korea; 4 Animal Genomics and Bioinformatics Division, National Institute of Animal Science, Wanju, Jeonbuk Republic of Korea; 5 Department of Psychiatry, Seoul National University Bundang Hospital, 173-82, Gumi-ro, Bundang-gu, Seongnam, Gyeonggi-do 463-707 South Korea; 6 Department of Psychiatry, College of Medicine, Seoul National University, Seoul, Republic of Korea

**Keywords:** Autism spectrum disorder, Arginine vasopressin receptor 1A (AVPR1A), Microsatellite, Single nucleotide polymorphism, Association, Promoter

## Abstract

**Background:**

Arginine vasopressin has been shown to affect social and emotional behaviors, which is mediated by the arginine vasopressin receptor (AVPR1A). Genetic polymorphisms in the *AVPR1A* promoter region have been identified to be associated with susceptibility to social deficits in autism spectrum disorder (ASD). We hypothesize that alleles of polymorphisms in the promoter region of *AVPR1A* may differentially interact with certain transcriptional factors, which in turn affect quantitative traits, such as sociality, in children with autism.

**Methods:**

We performed an association study between ASD and polymorphisms in the *AVPR1A* promoter region in the Korean population using a family-based association test (FBAT). We evaluated the correlation between genotypes and the quantitative traits that are related to sociality in children with autism. We also performed a promoter assay in T98G cells and evaluated the binding affinities of transcription factors to alleles of rs7294536.

**Results:**

The polymorphisms—RS1, RS3, rs7294536, and rs10877969—were analyzed. Under the dominant model, RS1–310, the shorter allele, was preferentially transmitted. The FBAT showed that the rs7294536 A allele was also preferentially transmitted in an additive and dominant model under the bi-allelic mode. When quantitative traits were used in the FBAT, rs7294536 and rs10877969 were statistically significant in all genotype models and modes. Luciferase and electrophoretic mobility-shift assays suggest that the rs7294536 A/G allele results in a Nf-κB binding site that exhibits differential binding affinities depending on the allele.

**Conclusion:**

These results demonstrate that polymorphisms in the *AVPR1A* promoter region might be involved in pathophysiology of ASD and in functional regulation of the expression of *AVPR1A*.

**Electronic supplementary material:**

The online version of this article (doi:10.1186/s13229-017-0161-9) contains supplementary material, which is available to authorized users.

## Background

Autism spectrum disorder (ASD) is complex neuropsychiatric developmental disorders characterized by social communication impairments and restricted interests [1]. In spite of heterogeneous etiology, molecular genetic studies for ASD have identified susceptible candidate genes, such as those involved in neurobiological pathways, chromatin remodeling, protein translation, actin dynamics, and synaptic functions [2, 3].

Arginine vasopressin receptor 1A (AVPR1A), a receptor for arginine vasopressin (AVP), is coupled to the G_αq/11_ G-protein coupled receptor and is activated by binding AVP via phospholipase C. The subsequent activation of protein kinase C helps mediate neuronal development, memory formation, synaptic plasticity, and neuronal survival [4, 5]*.* Animal and human physiobiological studies have also demonstrated that social behavior is affected by AVPR1A [6–8]. Furthermore, AVP and AVPR1A have been shown to play crucial roles in the pathophysiology of psychiatric disorders such as anxiety, depression, and post-traumatic stress disorder in rodents [9, 10]. AVPR1A signaling has also been shown to be involved in human social and emotional behavior as demonstrated in a study using the AVPR1A antagonist SRX246 [9]. Furthermore, administration of AVP via a nasal spray has been shown to improve social recognition of emotionally balanced faces in humans [11]. The variations in the gene structure of *AVPR1A* mostly occur in the form of genetic polymorphisms, which have been proposed to underlie inter- and intra-specific differences in expression levels [12, 13] and spatial expression patterns in the brain. The promoter region of *AVPR1A* includes a number of highly polymorphic microsatellites, such as RS1 and RS3 [14]. Furthermore, genetic variations in the promoter region of *AVPR1A* have also been associated with risks for autism, in which social deficits are the major symptoms, as well as autism phenotypes in nonclinical populations [15–19]. Previous association studies from various populations have been focused on examining association of RS 1, RS3, and a few SNPs resulting in inconsistent results [20]. For instance, Tansey et al. [13] demonstrated that the shorter alleles of RS1 had weaker associations with autism in the Irish population. One recent study reported association of RS1 and RS3 with repetitive behaviors, but not diagnosis of ASD [21]. Both RS1 and RS3 showed differences in relative promoter activity as measured in the human neuroblastoma cell line, SH-SY5Y, with the shorter repeat alleles of RS1 and RS3 exhibiting decreased relative promoter activity [13]. Meyer-Lindenberg et al. [22] also reported abnormal amygdala signaling is involved in the pathophysiology of autism. Our previous studies have also shown statistically significant associations between ASD and the shorter alleles of RS1 and RS3, which have been reported to be over-transmitted as risk alleles in other populations [23]. We have also previously reported a statistically significant association of ASD with the single nucleotide polymorphisms (SNPs) rs10877969 and rs7294536 in the promoter region of *AVPR1A* [24].

Overall, the first objective of this study was to validate our previous association study between ASD and the polymorphisms RS1, RS3, rs7294536, and rs10877969 in *AVPR1A* with samples from independent cohorts from our previous studies. We analyzed quantitative correlation between genotypes and quantitative traits in probands with ASD. For these analyses, we selected quantitative traits which reflect sociality directly among various inventories used for phenotypic measures of the subjects. The second is to determine the effects of the polymorphisms on the expression level of *AVPR1A*. Therefore, we used luciferase assays to ascertain the functional sequence polymorphisms in the promoter region in T98G cells. We also use electrophoretic mobility-shift assay (EMSA) to evaluate how the promoter polymorphisms affect nuclear protein-binding affinities. For this, we analyzed Korean family trios composed of subjects with ASD and their biological parents, diagnosed by pervasive developmental disorder based on DSM-IV-TR diagnostic criteria.

## Methods

### Subjects

Probands and their biological parents were ascertained for ASD based on the DSM-IV-TR diagnostic criteria. To confirm the ASD diagnosis, the parents or caregivers were interviewed with the Korean version of Autism Diagnostic Interview-Revised (K-ADI-R) [25], and the probands were assessed with the Korean version of the Autism Diagnostic Observation Schedule (K-ADOS) [26]. We assessed “social ability and other behavioral symptoms of the probands by using the Social Communication Questionnaires (SCQ) [27], Asperger Syndrome Diagnostic Scale (ASDS) [28], Social Responsiveness Scale (SRS) [29], and Korean Child Behavior Checklist (K-CBCL) [30].” The Korean versions of the Social Maturity Scale (K-SMS) [31], Vineland Adaptive Behavior Sales-Interview (VABS) [32], and of Korean Educational Development Institute-Wechsler Intelligence Scale for Children-Revised (KEDI-WISC-R) [33] were used for examining adaptive functioning and intelligence. We only included subjects showing clear phenotype of ASD by clinical best estimate diagnosis by two board certified psychiatrists, based on those diagnostic instruments and measures. For diagnostic validity and reliability, the age of the probands was limited to 36 months or older. We did not put any limitation for gender. For the probands suspected to have known genetic or neurological anomalies, including Down syndrome, fragile X syndrome, tuberous sclerosis, and karyotyping and neurological examinations were performed in the screening stage to identify and exclude those subjects from further analyses. The subjects either parent has declared he or she has non-Korean ethnicity were excluded from enrolment. Written informed consent was obtained from the biological parents or caregivers. This study was approved by Seoul National University Bundang Hospital Institutional Review Board (IRB_B-1406/253–001).

### Genotyping

Blood samples in EDTA tubes were kept at −70 °C before use. Genomic DNA was extracted from blood using QIAamp^®^ DNA Blood kit (Qiagen, Seoul, Korea). Amplifications of the RS3 [Complex (CT)_4_-TT-(CT)_8_-(GT)_24_] and RS1 [(GATA)_*n*_ tetranucleotide repeats] were performed according to method reported by Yang et al. (2010) (Additional file 1: Table S1 shows this in more detail). The size of the labeled PCR products was determined by capillary electrophoresis on an ABI 3100 sequencer using Gene Scan Software 2.02 (ABI, Foster city, CA, USA). Five alleles of RS3 were found in all participants in multiples of 2 bp, and there were six alleles of RS1 with multiples of 4 bp.

Two SNPs, rs7294536 and rs10877969, were genotyped using sets of primers and probes (BMS, Seoul, Korea) (Additional file 1: Table S1 shows this in more detail). PCRs were performed with Real-time Master Mix (Toyobo, Osaka, Japan) following proper reaction conditions on the real-time PCR machine (BMS, Seoul, Korea). The genotyped data was collected and the Mendelian inheritance errors for each individual polymorphism were checked by PedCheck (v.1.1) to verify the data quality and to identify any genotyping error. Detail procedure was descripted in Additional file 1: Supplementary Materials and Methods.

### Luciferase assay

We used *Tfsitescan* (http://www.ifti.org/) to predict rs7294536 variants present within the Nf-κB consensus sequence (5′-GGGRNNYYCC-3′). A 1641 bp fragment encompassing −108 to −1749 bp from of the 5′ regulatory region of *AVPR1A* was amplified via PCR using human DNA (Additional file 1: Table S1 shows this in more detail). The primers used for cloning *AVPR1A* contained *Kpn*I and *Bgl*II linkers. The PCR products were first digested by *Kpn*I and *Bgl*II and then were cloned into the multiple cloning site of the pGL3 reporter vector (Promega, Madison, WI, USA). The rs7294536 A/G (−1502 bp) and rs10877969 A/G (−649 bp) variants were generated using the QuickChange^®^ Site-Directed Mutagenesis Kit (Stratagene, LA Jolla, CA, USA) with each primer set (Additional file 1: Table S1 shows this in more detail). For all four haplotype constructs, the pA-A construct was subsequently used as a template to generate additional possible haplotype constructs (i.e., pA-A, pA-G, pG-A, and pG-G). The pGL3-SV40-*AVPR1A* construct (encompassing −108 to −1749 bp from the 5′ regulatory region of *AVPR1A*), pRLCMV-*renilla*, pCMV-p65, and pGL3-basic vectors were used to transfect T98G cells, which is a human glioblastoma multiforme cell line, using FuGENE HD (Roche, Mannheim, Germany). Cell lysates were assessed using the Dual-Glo Luciferase Assay System (Promega, Madison, WI, USA) in a Lumat LB 9507 luminometer (EG &G Berthhold, Bad Wildbad, Germany). Further details are described in the Additional file 1: Supplementary Materials and Methods.

### EMSA and western blotting

EMSAs were used examine for any quantitative differences in the binding affinity of Nf-κB sub-family proteins to the A or G allele of rs7294536. A nuclear extract was prepared from transfected T98G cells that were either unstimulated or stimulated with 30 ng/mL TNF-α for 15, 30, and 180 min. Unlabeled double-stranded probes were annealed with equimolar concentrations of sense and antisense single-stranded probes. Radiolabeled probes were labeled with [α-^32^P] dCTP at the 2-base overhang (GG) on the 5′-end via end labeling with a Klenow fragment and then purified through a Sephadex G50 column. During the pre-incubation, the binding reaction was performed with the prepared nuclear extract and unlabeled probes in the reaction buffer at room temperature. Next, p50/p65 antibodies were added for the EMSA. Thereafter, radiolabeled probe (1 ng, 1 × 10^5^ cpm) was added to the reaction mixture and then incubated for 10 min at room temperature. The reaction mixtures were fractionated on a non-denaturing 6% polyacrylamide gel at 200 v for 2 h. The gel was transferred to 3MM paper, dried, and then was visualized by exposing it to X-ray film. For the EMSA, 10 μg of the nuclear proteins were denatured and separated on a 4–20% SDS-polyacrylamide gel (Invitrogen, Medison, WI, USA). The fractionated proteins were then transferred to a nylon membrane (Millipore, Bedford, MA, USA), and a western blot was performed with antibodies against rabbit p50 and p65 (Santa Cruz Biotechnology**,** Santa Cruz CA, USA). Further details are described in the Additional file 1: Supplementary Materials and Methods.

### Statistical analysis

To assess each individual polymorphism and haplotype, the transmission disequilibrium test (TDT) using the family-based association test (FBAT) program package (http://www.biostat.harvard.edu/fbat/fbat.htm) under available modes and models was used. The haplotype family-based association test (HBAT) from the FBAT program package was used to analyze associations between ASD and the haplotypes and ASD with a minimum frequency of 5%. A permutation test of 100,000 cycles was performed using the Monte Carlo option to determine the statistical power of HBAT. In addition, the *D*′ value between the microsatellites and SNPs was estimated with the FBAT program package. Statistical significance was considered at *p* < 0.05 for the TDT haplotype analysis. In addition to the basic TDT and HBAT, we measured multiple quantitative traits using the FBAT program. Quantitative traits include the total score of the ADI-R diagnostic algorithm, SCQ (lifetime), and subdomain scores of ASDS, SRS, and K-CBCL (social problems). All of the *p* values from the FBAT in our study were subject to Bonferroni correction to account for multiple testing.

The statistical analyses for luciferase assay were conducted using a Kruskal-Wallis test, followed by Dunn’s post hoc test or Mann-Whitney *U* test. Statistical significance was considered at *p* < 0.05. The relative luciferase units (RLUs) of the constructs were compared via analysis of variance (ANOVA) or unpaired Student’s *t* tests (SPSS. ver.15.0, Chicago, IL, USA).

## Results

### Subjects

A total of 212 families composed of affected children (*n* = 212), as well as their biological mothers (*n* = 212) and fathers (*n* = 209) were analyzed. The mean age of proband was 97.72 ± 56.42 months old (average ± standard deviation). Of the probands, 84.44% were males, 87.3% had autistic disorder, 11.3% had pervasive developmental disorder not otherwise specified (PDD-NOS), and 1.4% had Asperger’s syndrome as defined by the DSM-IV-TR diagnostic criteria. Clinical data of the probands for sociality traits are summarized in Additional file 1: Table S2.

### Genetic association study of the RS1 and RS3 microsatellite polymorphisms

No associations were observed between RS3 and ASD in the present replication sample set, though a significant association was observed in our previous analyses [23]. The shot allele, RS1–310, was preferentially transmitted in the dominant model under the allelic mode (*Z* = 2.302; *p* = 0.021) (Table 1).

**Table 1:** Family-based association test of polymorphisms in *AVPR1A* promoter region

*Marker*	*Bi-allelic mode*	*Model*	*Additive*			Dominant			Recessive		
	*Allele*	*Freq.*	*N*	*Z*	*p*	*N*	*Z*	*p*	*N*	*Z*	*p*
RS3	326	0.062	38	− 0.617	0.537	37	− 0.714	0.475	*–*	*–*	*–*
	328	0.207	96	0.183	0.855	94	− 0.464	0.643	21	0.612	0.107
	330	0.255	104	− 0.254	0.799	95	− 0.981	0.327	37	1.172	0.241
	332	0.202	97	1.044	0.296	91	1.272	0.203	18	− 0.25	0.803
	334	0.186	98	0.447	0.655	94	− 0.621	0.535	25	0.221	0.825
rs7294536	A	0.807	112	2.062	0.039	36	2.043	0.041	*36*	*− 2.043*	0.041
	G	0.193	112	− 2.062	0.039	107	− 1.386	0.166	*–*	*–*	*–*
RS1	306	0.087	59	0.367	0.714	58	0.131	0.896	107	1.386	0.166
	310	0.414	152	1.490	0.136	122	2.302	0.021	86	− 0.288	0.773
	314	0.221	124	− 1.121	0.262	119	− 0.323	0.747	31	− 2.090	0.037
	318	0.083	62	− 1.543	0.123	61	− 1.440	0.150	*–*	*–*	*–*
	322	0.057	39	0.000	1.000	39	0.155	0.877	*–*	*–*	*–*
	326	0.130	77	0.211	0.833	76	0.349	0.727	13	− 0.316	0.752
rs10877969	A	0.911	68	4.796	0.001	18	2.177	0.030	67	4.573	< 0.001
	G	0.089	68	− 4.796	0.001	67	− 4.573	< 0.001	18	− 2.177	0.030
	*Multi-allelic mode*		*Df*	*χ* ^*2*^	*p*	*Df*	*χ* ^*2*^	*p*	*Df*	*χ* ^*2*^	*p*
RS3			5	1.598	0.902	5	4.436	0.489	4	4.133	0.388
rs7294536			1	4.525	0.039	2	5.377	0.068	2	5.377	0.068
RS1			6	4.517	0.607	6	8.425	0.209	3	4.634	0.201
rs10877969			1	23.000	< 0.001	2	23.206	< 0.001	2	23.206	< 0.001

*Allele* over-transmitted allele, *Freq*. frequency, *N* number of informative nuclear families, *Z Z* score from family-based association test, *Df* degree of freedom, *χ* ^*2*^ *χ* ^*2*^ statistics, *p*, *p* value

### Genetic association study of single nucleotide polymorphisms

The FBAT showed that the rs10877969 A allele was significantly transmitted in all models and modes (*Z* = 4.796; *p* < 0.001). The rs7294536 A allele also showed significant transmission in the additive (*Z* = 2.062; *p* = 0.039) and dominant (*Z* = 2.043; *p* = 0.041) models in the bi-allelic mode. These findings were consistent with our previous report regarding the transmission of *AVPR1A* SNPs in ASD (Table 1).

### Haplotype analyses

Haplotypes were selected from different combinations of the four polymorphisms (RS1, RS3, rs7294536, and rs10877969) using a sliding window approach (frequency < 0.05). A significant association was found in two haplotypes of rs7294536/rs10877969, A/A (*p* < 0.001) and A/G (*p* < 0.001), under the bi-allelic mode. The RS1/rs10877969 (310-A, *p* = 0.011) and rs7294536/RS1/rs10877969 (A-310-A, *p* = 0.012) haplotype combinations were significantly associated with ASD. However, there were no statistically significant associations between any RS1/RS3 haplotype combinations in this study (Table 2).

**Table 2:** Haplotype analysis of polymorphisms in promoter region of *AVPR1A*

*Marker*	Biallelic mode	Model	Additive			Dominant			Recessive		
	Haplotype	Freq.	*N*	*Z*	*p*	*N*	*Z*	*p*	*N*	*Z*	*p*
1-3	330-310	0.135	70	− 0.753	0.452	69	− 0.770	0.441	11	− 0.164	0.869
2-4	A-A	0.736	133	3.826	< 0.001	55	3.651	< 0.001	118	2.655	0.001
	A-G	0.064	48	− 3.586	< 0.001	48	− 3.311	< 0.001	–	–	–
1-2	330-A	0.235	109	− 0.230	0.818	99	− 0.937	0.349	33	1.212	0.225
2-3	A-310	0.384	145	1.639	0.101	116	2.112	0.035	72	0.125	0.900
3-4	310-A	0.367	149	2.552	0.011	124	2.995	0.003	72	0.564	0.573
1-2-3	330-A-310	0.125	65	− 0.891	0.373	65	− 1.045	0.296	10	0.174	0.862
2-3-4	A-310-A	0.352	142	2.505	0.012	118	2.650	0.008	61	0.878	0.380
	Multi-allelic mode		Df	χ^2^	*p*	Df	χ^2^	*p*	Df	χ^2^	*p*
1-3			6	6.538	0.366	6	5.425	0.491	–	–	–
2-4			3	24.302	< 0.001	3	20.364	< 0.001	2	9.650	0.008
1-2			6	3.859	0.696	6	3.779	0.707	3	6.899	0.075
2-3			5	5.594	0.348	5	7.474	0.188	2	2.148	0.341
3-4			5	18.501	0.002	5	18.934	0.002	2	4.027	0.134
1-2-3			6	7.455	0.281	6	6.676	0.352	–	–	–
2-3-4			5	19.869	0.001	5	17.603	0.003	2	2.612	0.271

*1* RS3 (complex), *2* rs7294536, *3* RS1 (GATA), *4* rs10877969, *N* number of informative nuclear families, *Df* degree of freedom, *χ* ^*2*^ *χ* ^*2*^ statistics, *p p* value

### Quantitative association of the markers

The FBAT analysis showed significant association between the SNPs (rs7294536 and rs10877969) and multiple quantitative traits related to sociality and social communication, including total scores of SCQ and SRS, social trait score of ASDS, and subdomain scores of the “qualitative abnormality in reciprocal social interaction” domain of ADI-R (*p* = 0.03 to < 0.001). For rs10877969, the FBAT analysis for quantitative traits was statistical significant in all models and modes. Strongly significant results (*p* < 0.001) were observed for analyses of ASDS social, SRS total, social problems in K-CBCL social problems, ADIR-A1: failure to use nonverbal behaviors to regulate social interaction, and VABS-social ability (Table 3). Furthermore, rs7294536 was weakly associated with quantitative traits (*p* = 0.02–0.045). Weak associations were observed between the social phenotypes and either microsatellite polymorphisms. In the recessive model under the bi-allelic mode, the RS3–328 allele only showed weak significance with quantitative social phenotype scores (*p* = 0.02–0.036) (Additional file 1: Table S3 shows this in more detail).

**Table 3:** Family-based association results of polymorphisms in *AVPR1A* with single quantitative trait by additive model

Traits			SCQ	ASDS	SRS	K-CBCL	ADIR-A1	ADIR-A2	ADIR-A3	ADIR-A4	ADIR-total	VABS
Marker	Freq.	*N*	*p*	*p*	*p*	*p*	*p*	*p*	*p*	*p*	*p*	*p*
rs7294536 G	0.193	71	0.049	0.019	0.033	0.015	0.016	0.029	0.030	0.019	0.021	0.407
rs7294536 A	0.807	71	0.049	0.019	0.033	0.015	0.016	0.029	0.030	0.019	0.021	0.407
rs10877969 G	0.089	47	0.001	< 0.001	< 0.001	< 0.001	0.001	0.001	0.001	0.002	0.001	< 0.001
rs10877969 A	0.911	47	0.001	< 0.001	< 0.001	< 0.001	0.001	0.001	0.001	0.002	0.001	< 0.001

*Freq.* allele frequency, *N* number of informative nuclear families, *p p* value, *SCQ* Social Communication Questionnaires, *ASDS* Asperger Syndrome Diagnostic Scale, *SRS* social responsiveness scale, *K-CBCL* Korean Child behavior checklist (social problems), *K-ADIR* Autism Diagnostic Interview-Revised, *ADI-A1* failure to use nonverbal behaviors to regulate social interaction, *ADI-A2* failure to develop peer relationship, *ADI-A3* lack of shared enjoyment, *ADI-A4* lack of socioemotional reciprocity, *VABS* Vineland Adaptive Behavior Scales

Haplotype analyses of the quantitative traits for the RS1/SNP and SNP/SNP combinations were statistical significant. A significant effect was observed between the A/A and A/G haplotypes of rs7294536/rs10877969 for quantitative traits related to sociality in all models and modes (*p* < 0.05). The haplotype rs7294536/RS1/rs10877969 (A/310/A) and social traits were significantly associated in the additive model under multi-allelic mode (*p* < 0.001). Results for the A/G haplotype of rs7294536/rs10877969 was also significantly associated with social traits (*p* = 0.001–0.025).

A multi-trait test was performed for multiple traits (SCQ, ASDS, SRS, K-CBCL social problems, and VABS in bi-allelic mode and multi-allelic mode) and the rs7294536 and rs10877969 SNPs. The results from all models under the bi-allelic mode were statistically significant (additive model, *p* = 0.009; dominant model, *p* = 0.015; and recessive model, *p* = 0.015) (Additional file 1: Table S4 shows this in more detail). In the recessive model analysis for the A allele, variations of ADI-A4 measured between the AG/GG allele and AA allele had statistical significant differences (*t* = 2.434; *p* = 0.016) (Additional file 1: Table S5 shows this in more detail).

### Luciferase assay

**Fig. 1:**
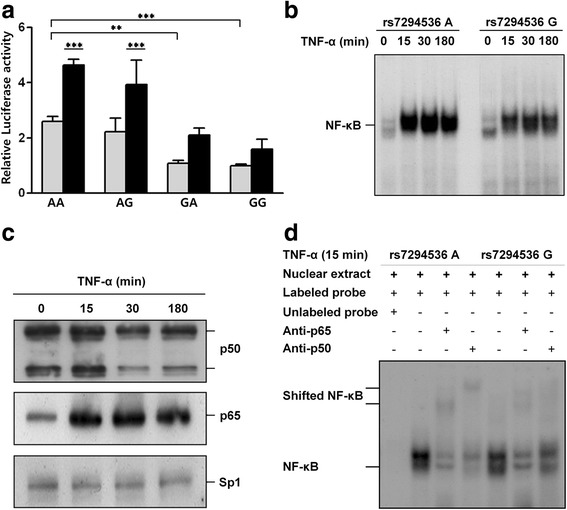
Luciferase assays and electrophoresis mobility-shift assay (EMSA). **a** Luciferase assays with vector constructs which were contained polymorphic sites, rs7294536 (−1502 A/G) and rs10877969 (− 649 A/G) in promoter region of the *AVPR1A*. The RLU values were denoted by mean and error bars by ± SD (*n* = 6). ***p < 0.001, **p < 0.01. **b** EMSA with oligonucleotides containing the rs7294536A or rs7294536G allele, **c** Western blotting with nuclear extract of T98G induced by 30 ng/ml of TNF-α. **d** Supershift assays with p65 and p50 antibodies and nuclear extract induced by 30 ng/ml TNF-α for 15 min

The relative luciferase activity for the promoter with the rs7294536 A allele was significantly higher than that of the G allele. However, rs10877969 allelic variants did not influence promoter activity (Fig. 1a).

### Analyses of transcriptional factor binding site

To investigate whether the putative Nf-κB involvement is due to a transcriptional event in the *AVPR1A* promoter region, we synthesized 22-mer oligonucleotide probes with adjacent sequences of rs7294536 and then performed an EMSA with radiolabeled DNA. From the luciferase assays, the promoter activities of each construct were measured under normal conditions without the activation of any pathways. Therefore, first, EMSA was performed with the nuclear extracts of 1, 3, 5, 10, and 20 μg in un-induced conditions for the Nf-κB pathway. We observed that the probe containing the rs7294536 G allele had a stronger binding affinity to unknown and nonspecific proteins than the A allele. To induce the Nf-κB pathway, the cells were treated with TNF-α (30 ng/ml) for 0, 15, 30, or 180 min. The nuclear extracts of the cells that were stimulated for 30 min showed the strongest band intensity. Thereafter, the nuclear proteins from this time point were used. Stimulating the cells with TNF-α resulted in a significant time-dependent increase in binding between the nuclear proteins and the probe when compared with the unstimulated cells. The binding of nuclear proteins to probes for the rs7294536 A and G alleles were discriminatively detected by EMSA. A higher intensity band was observed for the probe containing the rs7294536 A allele than the rs7294536 G allele (Fig. 1b). To verify the quantity of nuclear proteins used in the EMSA, we performed a western blot analysis for p50 and p65, and SP-1 was used as an internal control. The levels of p65 and p50 in the nuclear extract of the cells stimulated with TNF-α for 15 and 30 min were increased when compared to the unstimulated cells, and SP-1 was detected uniformly (Fig. 1c). To confirm the specificity for the binding of nuclear proteins to each probe, a competition experiment was performed by mixing the nuclear proteins with anti-p50 and anti-p65 antibodies before treating them with the labeled probes. We observed supershifted bands for p65 and p50 in the competition assay (Fig. 1d). Overall, this study suggests that the rs7294536 A/G polymorphisms affect the Nf-κB binding site affinity. Furthermore, these results demonstrate that SNPs in the promoter region of *AVPR1A* are indeed involved in regulating the expression of the *AVPR1A*.

## Discussion

Though comparisons between *AVPR1A* microsatellites and social behaviors have been previously reported in animal models, the associations of specific markers with ASD have not yet been replicated. Results from a previous association study between families with ASD and RS1 and RS3 in *AVPR1A* have been inconsistent with our previous study [23] as well as others’ studies. Kim et al. [15] first found nominally significant transmission disequilibrium of 332 bp in RS3 in 115 non-ethnic-matched ASD families but not by a low power in Bonferroni correction. Next, Wassink et al. [16] replicated an association test between the RS1 and RS3 microsatellites in Caucasian families with autism and found a strong transmission disequilibrium for 328 bp in RS3 and RS1-(GATA)_9_ in the “normal language” subgroup families. Yirmiya et al. [17], who studied 114 Israeli families, found a significant association with moderate LD between ASD and new markers located in the intron of *AVPR1A*.

The differences between these studies in regard to preferentially transmitted alleles may be due to the different ethnic groups that were studied and the varied statistical methodologies or sample size. In this study, our failure to replicate the genetic associations between microsatellite markers in *AVPR1A* and Korean individuals with ASD from our original study could be due to a several reasons. Firstly, our original findings might be false positives from lack of replication. Next, it can be possible that there is lack of adequate power to detect or replicate an association result because of lack of adequate sample size for the analysis of multiple alleles. We also cannot rule out the possibility that microsatellites frequently inherited by de novo alterations can also affect susceptibility [34]. Moreover, clinical heterogeneity of ASD must be considerable factor for replication failure.

Previously, we have suggested that microsatellites and SNPs of *AVPR1A* might be genetically involved in ASD [23, 24]. Moreover, this study also suggested that the social trait values among families with ASD were significantly associated with their genotypes. To the best of our knowledge, there has been no other replication study for *AVPR1A* polymorphisms in independent cohorts within the same population. This family-based association study replicated the results that suggested rs7294536 G and rs10877939 G alleles of *AVPR1A* have been over-transmitted as risk allele in families with ASD. A significantly low *p* value for rs10877939 and a moderate *p* value for rs7294536 in the FBAT were observed. These results are consistent with previous reports with other ethnicities. Tansey et al. [13] showed an association between autism and the SNPs in the *AVPR1A* promoter, demonstrating a single association with *AVPR1A* in an Irish autism trio collection with a corrected *p* value of 0.01 for rs11174815, a tag SNP located 143 bp downstream from rs10877969. However, this group concluded that a lack of a relationship between this marker and autism in the Irish autism sample population was probably due to the very low minor allele frequency (MAF = 0.015).

In the pathogenesis of ASD, it is assumed that multiple gene variants may be involved, each making small quantitative contributions to the final phenotype [35]. Reciprocal social interaction is one of the representative final phenotypes of ASD that is affected by genetic variants. In this study, two SNPs in *AVPR1A* showed significant quantitative associations with multiple measures of social behaviors and reciprocity both in current (ASDS, SRS, and VABS) and past developmental periods (ADI-R and SCQ), similar to that reported by Yirmiya et al. [17]. It also supports the notion that polymorphisms in the promoter region of *AVPR1A* might contribute to the dysregulation of social behavior, rather than the development of ASD itself, though studies in this area has been largely inconsistent between studies [36–39].

In addition, the functional role of the RS1 and RS3 microsatellites in *AVPR1A* has also been previously demonstrated by luciferase assays [13]. The shorter alleles of RS1 were hypothesized to increased susceptibility to autism as a result of decreased AVPR1A expression. However, compared to microsatellites, few studies have studied the function of SNPs in this region. Here, we investigated the potential role of the rs7294536 and rs10877969 SNPs in the promoter region by examining their effect on relative promoter activity and transcriptional factor interactions. We focused on rs7294536 since it is located in the recognition sequences for the Nf-κB transcription factors, which is expected to bind with p65. The construct with the rs7294536 A allele showed higher relative promoter activity, especially when Nf-κB pathway was overexpressed. Through the EMSA, it was confirmed that the Nf-κB binding affinities varied according to the rs7294536 alleles.

In our previous study, the rs7294536 A allele was over-transmitted in families with ASD and was considered a risk allele for ASD [24]. However, the rs7294536 A allele was observed to have higher relative promoter activity, which was counter to our hypothesis that lower promoter activity was related to autism-related deficits of behavior [12, 13]. This may be due varying *AVPR1A* expression levels and transfection efficiencies in the T98G cells used in this study. The T98G cell line is non-neuronal since it originated from human glioblastoma multiforme. Therefore, this can result in experimental inconsistencies with our previous study where the SH-SY5Y cell line was used instead.

Secondly, our experimental setup for the promoter activity study is reminiscent of inflammatory situations [40, 41]. The expression of IL-1β, IL-6, IL-17, and TNF inflammatory molecules is increased in the brain, cerebrospinal fluid, and serum of some patients with ASD, whereas NF-kB is activated in the brain, which then stimulates peripheral blood immune cells in patients with autism [42–44]. Nf-κB has been recognized as a member of the Rel transcription factor family and the most widely studied form of NF-κB is a heterodimer of the p50 and p65 subunits, which functions as a potent activator of gene transcription [40]. Moreover, neurons, astrocytes, and microglia from patients with ASD revealed higher expression of the p65 subunit of NF-κB as compared with matched controls. Therefore, NF-κB signaling was prominent in the interacting gene networks that were constructed from the brains of patients with ASD [45]. We hypothesized that elevated NF-κB might result in the protection of neuronal connectivity and signaling network due to the elevated AVPR1A expression. In this study, increased differences in the relative promoter activities of the rs7294536 A and rs7294536 G alleles were observed when cells overexpressed Nf-κB or treated with TNF-α as compared with untreated condition. This was also observed via EMSA by the differences in binding affinities between the rs7294536 A and rs7294536 G alleles.

This study could provide novel insight into the transcriptional regulation of *AVPR1A* and the potential biological roles of its gene product. Therefore, the role of *AVPR1A* polymorphisms should be explored with regard to the neurobiology of ASD, especially in the context of NF-κB signaling pathway. To investigate its molecular biological aspects, the expression pattern for the rs7294536 A/G alleles in *AVPR1A* in several cell types and specific brain regions of different organisms will be required. Furthermore, AVPR1A expression in the brain, the related polymorphic alleles, and subsequent activation of NF-κB signaling during inflammation will be needed to corroborate this study.

Although single predictable genetic markers were uninformative, multiple reports with accumulating evidence have demonstrated genetic associations with ASD [46]. Moreover, the carefully selected sets of critical genes should be able to be applied to sub-groups of ASD as classified by age, sex, severity, and phenotypic traits [47]. Overall, we identified a functional polymorphic site in *AVPR1A* that may be possibly important for socialization in ASD, and evidence was provided for its mechanistic biological role in the present study. The limitations of this study are; first, we only assessed two polymorphisms that were frequently observed in patients with ASD in this study. However, it is also possible that other undetected polymorphisms may have overt function with rs7294536 in the LD region, as there may be high LD with variants not tested. Second, the reason for inconsistency in the association analyses in microsatellite polymorphisms between current study and our previous report are not fully explained. Lastly, as the behavioral characteristics of the parents are not evaluated, there was limitation in examining relationship between *AVPR1A* variants and behavioral functioning in the context of broader autism phenotype.

## Conclusion

In this family-based association study, we validated our previous study between ASD and the polymorphisms in *AVPR1A* with independent samples. Also, we observed quantitative correlation between genotypes and quantitative traits in probands with ASD. Luciferase and electrophoretic mobility-shift assays exhibited differential binding affinities depending on the allele of associated SNP. These results demonstrate that polymorphisms in the *AVPR1A* promoter region might be involved in pathophysiology of ASD and in functional regulation of the expression of *AVPR1A*.

## Additional file

Additional file 1: Supplementary Materials and Methods, **Table S1**, **Table S2**, **Table S3**, **Table S4**, **Table S5**, **Table S6**, and **Table S7**. Details of genotyping, luciferase assay, and electrophoretic mobility-shift assay are described as text. Supplementary tables include primers and probes used in this study (Table S1), description of social traits for ASD probands (Table S2), additional results of family-based association of polymorphisms in *AVPR1A* with single quantitative trait by additive model (Table S3), FBAT results with multi-trait such as SCQ, ASDS, SRS, K-CBCL, and VABS (Table S4), statistical analysis of the social behavior scores in subjects with ASD with genotypes for rs10877969 and rs7294636 (Table S5), genotype and allele frequency of rs10877969 in dbSNP b126 chr12:61,833,506..61833506 of various population (Table S6), and comparison of previous association studies for AVPR1A polymorphisms in ASD (Table S7).(DOCX 62 kb)

## Abbreviations

ADI-A1: Failure to use nonverbal behaviors to regulate social interaction
ADI-A2: Failure to develop peer relationship
ADI-A3: Lack of shared enjoyment
ADI-A4: Lack of socioemotional reciprocity
ANOVA: Analysis of variance
ASD: Autism spectrum disorder
ASDS: Asperger Syndrome Diagnostic Scale
AVP: Arginine vasopressin
AVPR1A: Arginine vasopressin receptor 1A
Df: Degree of freedom
EMSA: Electrophoretic mobility-shift assay
FBAT: Family-based association test
HBAT: Haplotype family-based association test
IRB: Institutional Review Board
K-ADI-R: Korean version of the Autism Diagnostic Interview-Revised
K-ADOS: Korean version of the Autism Diagnostic Observation Schedule
K-CBCL: Korean Child Behavior Checklist
KEDI-WISC-R: Korean Educational Development Institute-Wechsler Intelligence Scale for Children-Revised
K-SMS: Korean version of the Social Maturity Scale
MAF: Minor allele frequency
PDD-NOS: Pervasive developmental disorder that were not otherwise specified
RLUs: Relative luciferase units
SCQ: Social Communication Questionnaires
SNPs: Single nucleotide polymorphisms
SRS: Social Responsiveness Scale
TDT: Transmission disequilibrium test
VABS: Vineland Adaptive Behavior Sales-Interview
