# Supplementary material for: Replicative genetic association study between functional polymorphisms in AVPR1A and social behavior scales of autism spectrum disorder in the Korean population
Source: Mol Autism. 2017 Aug 9;8:44. doi: 10.1186/s13229-017-0161-9 (PMC5550983; doi:10.1186/s13229-017-0161-9)
Supplement: Supplementary file 1 — Supplementary Materials and Methods, Table S1, Table S2, Table S3, Table S4, Table S5, Table S6, and Table S7. Details of genotyping, luciferase assay, and electrophoretic mobility-shift assay are described as text. Supplementary tables include primers and probes used in this study (Table S1), description of social traits for ASD probands (Table S2), additional results of family-based association of polymorphisms in AVPR1A with single quantitative trait by additive model (Table S3), FBAT results with multi-trait such as SCQ, ASDS, SRS, K-CBCL, and VABS (Table S4), statistical analysis of the social behavior scores in subjects with ASD with genotypes for rs10877969 and rs7294636 (Table S5), genotype and allele frequency of rs10877969 in dbSNP b126 chr12:61,833,506..61833506 of various population (Table S6), and comparison of previous association studies for AVPR1A polymorphisms in ASD (Table S7).(DOCX 62 kb) [file 13229_2017_161_MOESM1_ESM.docx]

**Replicative genetic association study between functional polymorphisms in *AVPR1A* and social behavior scales of Korean autism spectrum disorders**

Supplemental Information

**Supplementary Material and Methods**

*Genotyping of Microsatellites and Single Nucleotide Polymorphism*

We performed an analysis for identifying microsatellites in the 5′ flanking region for RS3 and RS1. Amplification of the RS3-Complex (CT)_4_-TT-(CT)_8_-(GT)_24_ dinucleotide motif, located at 3,625 bp upstream of the transcription start site, was performed with the following set of primers: HEX-5′-TCCTGTAGAGATGTAAGTGC-3′ and reverse 5′-TCTGGAAGAGA CTTAGATGG-3′. Reaction of amplification was proceed by following condition; an initial denaturation at 94°C for 2 min, 35 cycles at 95°C (20 s), 55.5°C (10 s), 72°C (20 s), and a final extension step of 72°C for 2 min with maxime PCR premix (Intron, Daejeon, Korea).

PCR reaction of the RS1-(GATA)_n_ tetranucleotide repeats located at 553 bp upstream of the transcription start site were achieved using the following set of primers: forward FAM-5′-AGGGACTGGTTCTACAATCTGC-3′ and reverse 5′-ACCTCTCAAGTTATGTTGGTGG-3′. For PCR reactions, *AccuPower^®^* PCR PreMix (Bioneer, Daejeon, Korea) was used with 20 ng of genomic DNA and 10 pmol of each of the primers described above. The PCR conditions included an initial denaturation at 95°C for 5 min, followed by 35 cycles at 95°C (30 s), 55.5°C (15 s), 72°C (15 s), and a final extension step of 72°C for 2 min. The size of the labeled PCR products was determined by capillary electrophoresis on an ABI 3100 sequencer using Gene Scan Software 2.02 (ABI, Foster city, CA, USA). The number of repeats for the amplicons was identified by sequencing.

In our previous studies, rs10877969 and rs7294536 showed statistically significant association. These two SNPs were only used in this study. Each SNP sites were genotyped using sets of primers and probes (BMS, Seoul, Korea) for SNP, which were designed for genotyping assay. Primers and probes were presented in Supplemental table 1. The real-PCR reaction was performed in Realtime Master Mix (Toyobo, Osaka, Japan) followed proper reaction condition. The genotyped data were collected and Mendelian inheritance error for each individual polymorphism was checked by PedCheck (v.1.1) to check the data quality and genotyping error.

*Luciferase assay*

An 1,641bp fragment encompassing -108 to -1749 of the 5′ regulatory region of the *AVPR1A* was amplified by using human DNA with primers containing restriction endonuclease sites and cloned into a luciferase expression vector, pGL3 (Promega, Madison, WI, USA) between *Kpn*I and *Bgl*II. To amplify region including the polymorphic sites, microsatellites and SNPs, PCR template was used with genomic DNA of each subject identified through genotyping. Except for the polymorphic sites, the sequences amplified by PCR were identity. The primers used for cloning of the *AVPR1A* promoter region were forward, 5′-ATGCGGTACC TGCAGGGTTGGAATGTATCT-3′ and reverse, 5′-ATGCAGATCTTGGGTATTGCAAAA GTGGTT-3′ containing *Kpn*I and *Bgl*II linker (underlined sequences), respectively. The PCR reaction was performed with 20 ng, primers described above of 10pmol and *AccuPower*^®^ PCR PreMix (Bioneer, Daejeon, Korea) in a 20 μL volume. The PCR reaction was initially processed at 95°C for 5 min followed by 35 cycles of 95°C (45 sec), 58°C (45 sec), 72°C (2 min) and a final step of 72°C for 5 min. The PCR product digested by *Kpn*I and *Bgl*II were cloned into multicloning site of pGL3 reporter vector opened with same restriction enzymes.

Based on previous data of Tansey et al. (2011), the (GATA)_10_ repeat construct was used in mutagenesis process. The -1502 A/G (rs7294536) and -649 A/G (rs10877969) variants were generated using QuickChange^®^ Site-Directed Mutagenesis Kit (Stratagene, LA Jolla, CA, USA) and each primer set, *AVPR1A* (-1502)-F 5′-taccatggaaacCccccatttttttac-3′and *AVPR1A* (-1502)-R 5′-gtaaaaaaatggggG gtttccatggta-3′ for rs7294536 and *AVPR1A* (-649)-F 5′-ccagtccctttgtttaaCccatatagt-3′ and *AVPR1A* (-649)-R 5′-actatatggGttaaacaaagggactgg-3′ for rs10877969. For all four haplotype constructs, pA-A construct was subsequently used as template and generated possible haplotype constructs (i.e. pA-A, pA-G, pG-A, and pG-G).

The T98G; a human glioblastoma multiforme cells purchased from American Type Culture Collection were employed in this study. T98G cell lines were cultured in DMEM media supplemented with 10% heat-inactivated fetal bovine serum (Hyclone, **Waltham**, MA, **USA**). Cells in passage 5 - 10 were used for transfection. The cells (1×10^5^ - 5×10^5^) were plated, transfected with 0.8 μg pGL3-SV40-*AVPR1A* promoter constructs; pA-A, pA-G, pG-A, and pG-G for the SNPs and 0.2 μg pRLCMV-renilla plasmid using Fugene HD (Roche, Mannheim, Germany).

To identify the influence for the transcriptional regulation, the pRLCMV renilla plasmid that provide the constitutive expression of *Renilla* luciferase was used as an internal control to correct for differences in transfection and harvesting efficiency. After transfection, cells were incubated for 48hr. The preparation of cell lysate and assessment for luciferase activity was performed using Dual-Glo Luciferase Assay System (Promega, Madison, WI, USA) according to manufacturer’s manual. Luciferase activity was measured using a Lumat LB 9507 luminometer (EG &G Berthhold, Bad Wildbad, Germany). *Firefly* luciferase activity as *AVPR1A* promoter activity were normalized using the activity of *Renilla* luciferase and were expressed relative light unit (RLU). The pGL3-control (pSV40-*luc^+^*) and pGL3-Basic (without promoter) vector (Promega, MD, USA) were used as a positive and negative control, respectively. Transcription factor (TF) binding sites were predicted by the *Tfsitescan* (http://www.ifti.org/cgi-bin/ifti/Tfsitescan.pl). The rs7294536 were identified with Nf-κB sub-family binding site. To explore the effect of the transcriptional level by Nf-κB sub-family, the over-expression vector of p65 subunit (pCMV-p65) was co-transfected with SNP construct, respectively. Six independent triplicate experiments were performed and the results were represented graphically as a ratio of pGL3-Basic vector activity. The RLUs of constructs were compared by analysis of variance (ANOVA) or unpaired Student’s t-tests (SPSS. ver.15.0, Chicago, IL, USA).

*Electrophoretic mobility-shift assay*

We performed the electrophoretic mobility-shift assays (EMSA) to examine whether the proteins of Nf-κB sub-family bind to the polymorphic site of G allele or A allele of rs7294536. To prepare the nuclear extract, T98G cells which was un-stimulated and stimulated with 30 ng/mL TNF-α for 15, 30 and 180 minutes was harvested in 1.5 mL ice-cold PBS and washed with hypotonic buffer (10 mM HEPES pH 7.9, 1.5 mM MgCl_2_, 10 mM KCl, 0.5 mM PMSF and 0.5 mM DTT). To disrupt the cell membrane, resuspended cells with hypotonic buffer were added 0.5% NP40, homogenized thoroughly in Dounce homogenizer, centrifuged at 1,700 rpm for 10 min at 4^◦^C. The collected nuclear pellet was suspended with lysis buffer for nuclear membrane (5 mM HEPES pH 7.9, 26% glycerol, 1.5 mM MgCl_2_, 0.2 mM EDTA, 300 mM NaCl, 0.5 mM PMSF and 0.5 mM DTT). The nuclear extract was obtained by centrifuging at 24,000 ×g for 10 minutes at 4^◦^C and quantified by Bradford method (Biorad, Hercules, CA, USA). The sense and antisense probes (-1509 to -1490) for polymorphisms of rs7294536 respectively were as follow: rare allele (G), 5′-ggAATGGGGGGTTTCCATG GTA-3′ (sense) and 5′-ggTACCATGGAAACCCCCCATT-3′ (antisense); common allele (A), 5′-ggAATGGGGAGTTTCCATGGTA-3′ (sense) and 5′-ggTACCATGGAAACTCCCCATT-3′ (antisense). A double-strand probe were complementarily annealed with equimolar concentration and radiolabeled with [α-^32^P] dCTP at additional 2-base overhang (gg) in 5′end using Klenow end labeling method. The labeled probes were purified through Sephadex G50 column. Binding reaction were first performed at room temperature for 20 min with prepared nuclear extract (5-10 μg) in reaction buffer [20 mM HEPES at pH 7.9, 50 mM KCl, 0.1 mM EDTA, 1 mM DTT, 5% glycerol, 200 μg/mL BSA, and 2 μg of poly(dI-dC)·poly(dI-dC)]. Additionally, the competition assay was needed unlabeled probe (100 fold molar excess of labeled probe) and p50/p65 antibodies (4 μg). After pre-incubation, radiolabeled probes (1 ng, 1×10^5^ cpm) was added to the reaction mixture. Final reaction mixture was then incubated for 10 minutes at room temperature. The reaction samples were fractionated on a non-denaturating 6 % polyacrylamide gel at 200 v for 2 hr. The gel was transferred to 3MM paper, dried under vacuum and heat (80 ^◦^C), and then was visualized by exposing to X-ray film.

The nuclear proteins 10 μg used in EMSA was denatured and loaded by 4-20% gradient SDS-polyacrylamide gel (Invitrogen, Medison, WI, USA) for the electrophoresis. The fractionated protein was transferred to nylon membrane (Millipore, Bedford, MA, USA) was subjected to Western blot analysis with antibodies against rabbit p50 and p65. Sp1 was used for internal control (Santa Cruz Biotechnology, Santa Cruz, CA, USA). Following by incubation with horseradish peroxidase-conjugated secondary antibody, the antibody and antigen complex were detected by enhanced chemiluminescence, according to the manufacturer’s instruction (Pierce, Rockford, IL, USA).

Table S1 Primers and probes used in this study

| Experiments | Primers and Probes |
| --- | --- |
| Amplification of RS3 and RS1 | |
| RS3 | Forward 5'- ATC TGC TCT CCT GGT T-3' |
|  | Reverse 5'- AAA GTC CGC TTT CCT TTG TAT-3' |
|  | 5'- FAM^TM^/TGTTTAACCCATATAGTTTTAAACA-3' |
|  | 5'- HEX^TM^/ACCAGTCC TTTGTTTAATCCATAT-3' |
| RS1 | Forward 5'- GGAGAAGTGCTG TGT ATG C -3' |
|  | Reverse 5'- GTTGGAATGTATCTGGTTGAA -3' |
|  | 5'- FAM^TM^/ACCATGGAAACTCCCCA-3' |
|  | 5'- HEX^TM^/ATGGAAACCCCCCATTTTTTTAC-3' |
| SNP Genotyping of SNPs | |
| rs10877969 | Forward 5'- ATC TGC TCT CCT GGT T-3' |
|  | Reverse 5'- AAA GTC CGC TTT CCT TTG TAT-3' |
|  | 5'- FAM^TM^/TGTTTAACCCATATAGTTTTAAACA-3' |
|  | 5'- HEX^TM^/ACCAGTCC TTTGTTTAATCCATAT-3' |
| rs7294536 | Forward 5'- GGAGAAGTGCTG TGT ATG C -3' |
|  | Reverse 5'- GTTGGAATGTATCTGGTTGAA -3' |
|  | 5'- FAM^TM^/ACCATGGAAACTCCCCA-3' |
|  | 5'- HEX^TM^/ATGGAAACCCCCCATTTTTTTAC-3' |
| Luciferase assay for rs7294536 | |
| Cloning | 5′-ATGCGGTACCTGCAGGGTTGGAATGTATCT-3′ |
|  | 5′-ATGCAGATCTTGGGTATTGCAAAAGTGGTT-3′ |
| Mutagenesis | AVPR1A (-1502)-Forward 5′-taccatggaaacCccccatttttttac-3′ |
|  | AVPR1A (-1502)-R 5′-gtaaaaaaatggggGgtttccatggta-3′ |
|  | AVPR1A (-649)-F 5′-ccagtccctttgtttaaCccatatagt-3′ |
|  | AVPR1A (-649)-R 5′-actatatggGttaaacaaagggactgg-3′ |
| Electrophoresis mobility shift assay for rs7294536 | |
| A allele | 5′-ggAATGGGGAGTTTCCATGGTA-3′ |
|  | 5′-ggTACCATGGAAACTCCCCATT-3′ |
| G allele | 5′-ggAATGGGGGGTTTCCATGGTA-3′ |
|  | 5′-ggTACCATGGAAACCCCCCATT-3′ |

Table S2 Description of social traits for ASD probands

| Traits | ASD  Probands | Minimum | 25%  Percentile | Median | 75% Percentile | Maximum | Mean | Std. Dev | Std. Error | Lower  95% CI | Upper 95% CI |
| --- | --- | --- | --- | --- | --- | --- | --- | --- | --- | --- | --- |
| SCQ | 197 | 0 | 14 | 21 | 26 | 52 | 19.82 | 7.941 | 0.5658 | 18.70 | 20.93 |
| ASDS | 176 | 0 | 7 | 9 | 11 | 23 | 8.835 | 3.318 | 0.2501 | 8.342 | 9.329 |
| SRS | 185 | 0 | 74 | 92 | 109.5 | 168 | 92.39 | 27.93 | 2.053 | 88.34 | 96.45 |
| K-CBCL | 181 | 37 | 61 | 67 | 72 | 100 | 66.62 | 9.264 | 0.6886 | 65.27 | 67.98 |
| ADI-A1 | 201 | 0 | 3 | 4 | 6 | 6 | 4.234 | 1.572 | 0.1109 | 4.015 | 4.452 |
| ADI-A2 | 201 | 0 | 5 | 7 | 8 | 8 | 6.164 | 1.946 | 0.1385 | 5.891 | 6.437 |
| ADI-A3 | 201 | 0 | 4 | 5 | 6 | 6 | 4.980 | 1.273 | 0.08976 | 4.803 | 5.157 |
| ADI-A4 | 201 | 2 | 6 | 7 | 8 | 10 | 7.015 | 1.930 | 0.1361 | 6.746 | 7.283 |
| A-total | 201 | 3 | 19 | 24 | 26 | 30 | 22.39 | 5.426 | 0.3827 | 21.64 | 23.15 |
| VABS | 167 | 19 | 41 | 61 | 82 | 940 | 83.92 | 100.4 | 7.770 | 68.58 | 99.26 |

SCQ; Social Communication Questionnaires, ASDS; Asperger Syndrome Diagnostic Scale, SRS; social responsiveness scale, K-CBCL; Korean Child behavior checklist (Social Problems Subscale),, K-ADIR; Autism Diagnostic Interview-Revised, ADI-A1: Failure to use nonverbal behaviors to regulate social interaction, ADI-A2: Failure to develop peer relationship, ADI-A3: Lack of shared enjoyment, ADI-A4: Lack of socioemotional reciprocity, VABS; [Vineland Adaptive Behavior Scales](http://researchautism.net/glossary/190/vineland-adaptive-behavior-scales-(vabs))

| Traits |  |  |  | SCQ | ASDS | SRS | K-CBCL | ADIR | | | | | VABS |
| --- | --- | --- | --- | --- | --- | --- | --- | --- | --- | --- | --- | --- | --- |
|  |  |  |  |  |  |  |  | A1 | A2 | A3 | A4 | Total |  |
| Marker | Allele | Freq. | N | *p* | *p* | *p* | *p* | *p* | *p* | *p* | *p* | *p* | *p* |
| RS3 | 326 | 0.062 | 22 | 0.940 | 0.901 | 0.798 | 0.825 | 0.928 | 0.946 | 0.812 | 0.691 | 0.851 | 0.312 |
|  | 328 | 0.207 | 60 | 0.598 | 0.849 | 0.789 | 0.417 | 0.671 | 0.779 | 0.642 | 0.545 | 0.649 | 0.267 |
|  | 330 | 0.255 | 62 | 0.870 | 0.673 | 0.648 | 0.323 | 0.466 | 0.241 | 0.397 | 0.421 | 0.361 | 0.361 |
|  | 332 | 0.202 | 65 | 0.692 | 0.896 | 0.700 | 0.835 | 0.592 | 0.856 | 0.763 | 0.707 | 0.733 | 0.194 |
|  | 334 | 0.186 | 57 | 0.487 | 0.839 | 0.915 | 0.941 | 0.785 | 0.971 | 0.871 | 0.737 | 0.837 | 0.760 |
| rs7294536 | A | 0.807 | 70 | 0.049 | 0.019 | 0.033 | 0.015 | 0.016 | 0.029 | 0.030 | 0.019 | 0.021 | 0.407 |
|  | G | 0.193 | 70 | 0.049 | 0.019 | 0.033 | 0.015 | 0.016 | 0.029 | 0.030 | 0.019 | 0.021 | 0.407 |
| RS1 | 306 | 0.087 | 39 | 0.503 | 0.228 | 0.386 | 0.277 | 0.388 | 0.511 | 0.562 | 0.244 | 0.396 | 0.797 |
|  | 310 | 0.414 | 96 | 0.532 | 0.660 | 0.760 | 0.694 | 0.860 | 0.979 | 0.740 | 0.779 | 0.839 | 0.364 |
|  | 314 | 0.221 | 82 | 0.785 | 0.894 | 0.740 | 0.857 | 0.861 | 0.977 | 0.969 | 0.634 | 0.840 | 0.331 |
|  | 318 | 0.083 | 35 | 0.581 | 0.752 | 0.797 | 0.601 | 0.891 | 0.866 | 0.656 | 0.946 | 0.847 | 0.189 |
|  | 322 | 0.057 | 22 | 0.587 | 0.653 | 0.905 | 0.677 | 0.889 | 0.974 | 0.757 | 0.816 | 0.857 | 0.371 |
|  | 326 | 0.130 | 49 | 0.641 | 0.327 | 0.607 | 0.529 | 0.556 | 0.682 | 0.647 | 0.497 | 0.583 | 0.808 |
| rs10877969 | A | .911 | 46 | 0.001 | < 0.001 | < 0.001 | < 0.001 | 0.001 | 0.001 | 0.001 | 0.002 | 0.001 | < 0.001 |
|  | G | .089 | 46 | 0.001 | < 0.001 | < 0.001 | < 0.001 | 0.001 | 0.001 | 0.001 | 0.002 | 0.001 | < 0.001 |

Table S3 Family based association results of polymorphisms in *AVPR1A* with single quantitative trait by additive model

Freq.; allele frequency, N; number of informative nuclear families, *p*; p-value, SCQ; Social Communication Questionnaires, ASDS; Asperger Syndrome Diagnostic Scale, SRS; social responsiveness scale, K-CBCL; Korean Child behavior checklist (Social Problems Subscale), K-ADIR; Autism Diagnostic Interview-Revised, ADI-A1;Failure to use nonverbal behaviors to regulate social interaction, ADI-A2; Failure to develop peer relationship, ADI-A3; Lack of shared enjoyment, ADI-A4; Lack of socioemotional reciprocity, VABS; [Vineland Adaptive Behavior Scales](http://researchautism.net/glossary/190/vineland-adaptive-behavior-scales-(vabs))

Table S4 FBAT results with Multi-trait such as SCQ, ASDS, SRS, KCBCL and VABS

| Marker | Biallelic mode | Additive model | | |  | Dominant model | | |
| --- | --- | --- | --- | --- | --- | --- | --- | --- |
|  | Freq. | N | χ^2^ | p |  | N | χ^2^ | p |
| rs7294536 A | 0.807 | 72 | 9.947 | 0.077 |  | 23 | 3.671 | 0.598 |
| rs7294536 G | 0.193 |  |  |  |  | 70 | 10.335 | 0.066 |
| rs10877969 A | 0.911 |  |  |  |  | 14 | 3.543 | 0.617 |
| rs10877969 G | 0.089 | 48 | 15.424 | 0.009 |  | 47 | 14.178 | 0.015 |
|  | Multi allelic mode | Df | χ^2^ | p |  | Df | χ^2^ | p |
| rs7294536 |  | 5 | 15.424 | 0.009 |  | 10 | 13.356 | 0.204 |
| rs10877969 |  | 5 | 9.947 | 0.077 |  | 10 | 16.14 | 0.096 |

*Freq*., Frequency; N, number of informative nuclear families; *Df*, degree of freedom; *χ^2^, χ^2^* statistics; *p*, p-value

SCQ; Social Communication Questionnaires, ASDS; Asperger Syndrome Diagnostic Scale, SRS; social responsiveness scale, K-CBCL; Korean Child behavior checklist (Social Problems Subscale), VABS; [Vineland Adaptive Behavior Scales](http://researchautism.net/glossary/190/vineland-adaptive-behavior-scales-(vabs))

Table S5 Statistical analysis of the social behavior scores in subjects with ASD with genotypes for rs10877969 and rs7294636

|  |  |  | rs7294536 |  |  |  | rs10877969 |  |
| --- | --- | --- | --- | --- | --- | --- | --- | --- |
| Traits | Genotype | N | Average (±SEM) | Statistical value |  | N | Average (±SEM) | Statistical value |
| SCQ | AA | 129 | 20.220 (±0.637) | *t*=0.973 |  | 172 | 19.570  (±0.618) | *t*=1.501 |
|  | GA  GG | 68 | 19.060 (±1.109) | *p=*0.332 |  | 21 | 22.33  (±1.461) | *P=*0.135 |
| ASDS | AA | 115 | 8.983 (±0.322) | *t*=0.808 |  | 153 | 8.706 (±0.278) | *t*=1.508 |
|  | GA  GG | 61 | 8.557 (±0.392) | *P=*0.420 |  | 20 | 9.900 (±0.502) | *P=*0.133 |
| SRS | AA | 121 | 94.070 (±2.595) | *t*=1.126 |  | 161 | 91.120  (±2.248) | *t*=0.097 |
|  | GA  GG | 64 | 89.220 (±3.331) | *P=*0.262 |  | 21 | 102.00  (±5.001) | *P=*0.127 |
| K-CBCL | AA | 118 | 66.430 (±0.920) | *t*=0.381 |  | 158 | 62.630  (±0.732) | *t*=1.963 |
|  | GA  GG | 63 | 66.98  (±0.981) | *P=*0.704 |  | 19 | 62.630  (±2.214) | *P=*0.051 |
| ADI-A1 | AA | 131 | 4.405  (±0.137) | *t*=2.126 |  | 176 | 4.205 (±0.119) | *t*=1.279 |
|  | GA  GG | 70 | 3.914 (±0.183) | *P=*0.035 |  | 21 | 4.667 (±0.326) | *P=*0.202 |
| ADI-A2 | AA | 131 | 6.252 (±0.167) | *t*=0.866 |  | 176 | 6.114 (±0.154) | *t*=1.105 |
|  | GA  GG | 70 | 6.000 (±0.246) | *P=*0.388 |  | 21 | 6.619 (±0.300) | *P=*0.270 |
| ADI-A3 | AA | 131 | 4.977 (±0.114) | *t*=0.046 |  | 176 | 4.926 (±0.097) | *t*=1.545 |
|  | GA  GG | 70 | 4.986  (±0.146) | *P=*0.964 |  | 21 | 5.381 (±0.253) | *P=*0.124 |
| ADI-A4 | AA | 131 | 7.153 (±0.173) | *t*=1.387 |  | 176 | 6.926 (±0.148) | *t*=2.434 |
|  | GA  GG | 70 | 6.757  (±0.219) | *P=*0.167 |  | 21 | 8.000  (±0.293) | *P=*0.016 |
| ADI-total | AA | 131 | 22.790  (±0.484) | *t*=1.409 |  | 176 | 22.170  (±0.420) | *t*=1.997 |
|  | GA  GG | 70 | 21.66  (±0.618) | *P=*0.160 |  | 21 | 24.670  (±0.832) | *P=*0.047 |
| VABS | AA | 117 | 83.09 (±9.951) | *t*=0.164 |  | 142 | 88.800 (±9.040) | *t*=1.619 |
|  | GA  GG | 50 | 85.880 (±11.59) | *P=*0.870 |  | 22 | 51.410  (±4.489) | *P=*0.107 |

Freq.; allele frequency, N; informative probands, *p*; p-value, SCQ; Social Communication Questionnaires, ASDS; Asperger Syndrome Diagnostic Scale, SRS; social responsiveness scale, K-CBCL; Korean Child behavior checklist (Social Problems Subscale), K-ADIR; Autism Diagnostic Interview-Revised, ADI-A1;Failure to use nonverbal behaviors to regulate social interaction, ADI-A2; Failure to develop peer relationship, ADI-A3; Lack of shared enjoyment, ADI-A4; Lack of socioemotional reciprocity, VABS; [Vineland Adaptive Behavior Scales](http://researchautism.net/glossary/190/vineland-adaptive-behavior-scales-(vabs))

Table S6 Genotype and allele frequency of rs10877969 in dbSNP b126 [chr12:61833506..61833506](http://hapmap.ncbi.nlm.nih.gov/cgi-perl/gbrowse/hapmap28_B36/?name=chr12:61833506..61833506)

|  | Genotype frequencies | | | | | | | | | |  | Allele frequencies | | | | | | |
| --- | --- | --- | --- | --- | --- | --- | --- | --- | --- | --- | --- | --- | --- | --- | --- | --- | --- | --- |
|  |  |  |  |  |  |  |  |  |  |  |  | Ref allele | | | Other allele | | | |
| Population | geno-type | freq | count | geno-type | freq | count | geno-type | freq | count | Total |  | allele | freq | count | allele | freq | count | Total |
| ASW (A) | A/A | 0.211 | 12 | A/T | 0.509 | 29 | G/G | 0.281 | 16 | 57 |  | A | 0.465 | 53 | G | 0.535 | 61 | 114 |
| CEU (C) | A/A | 0.788 | 89 | A/T | 0.195 | 22 | G/G | 0.018 | 2 | 113 |  | A | 0.885 | 200 | G | 0.115 | 26 | 226 |
| CHB (H) | A/A | 0.721 | 98 | A/T | 0.243 | 33 | G/G | 0.037 | 5 | 136 |  | A | 0.842 | 229 | G | 0.158 | 43 | 272 |
| CHD (D) | A/A | 0.685 | 74 | A/T | 0.296 | 32 | G/G | 0.019 | 2 | 108 |  | A | 0.833 | 180 | G | 0.167 | 36 | 216 |
| GIH (G) | A/A | 0.356 | 36 | A/T | 0.515 | 52 | G/G | 0.129 | 13 | 101 |  | A | 0.614 | 124 | G | 0.386 | 78 | 202 |
| JPT (J) | A/A | 0.655 | 74 | A/T | 0.327 | 37 | G/G | 0.018 | 2 | 113 |  | A | 0.819 | 185 | G | 0.181 | 41 | 226 |
| LWK (L) | A/A | 0.174 | 19 | A/T | 0.477 | 52 | G/G | 0.349 | 38 | 109 |  | A | 0.413 | 90 | G | 0.587 | 128 | 218 |
| MEX (M) | A/A | 0.845 | 49 | A/T | 0.121 | 7 | G/G | 0.034 | 2 | 58 |  | A | 0.905 | 105 | G | 0.095 | 11 | 116 |
| MKK (K) | A/A | 0.115 | 18 | A/T | 0.442 | 69 | G/G | 0.442 | 69 | 156 |  | A | 0.337 | 105 | G | 0.663 | 207 | 312 |
| TSI (T) | A/A | 0.745 | 76 | A/T | 0.245 | 25 | G/G | 0.010 | 1 | 102 |  | A | 0.868 | 177 | G | 0.132 | 27 | 204 |
| YRI (Y) | A/A | 0.211 | 31 | A/T | 0.381 | 56 | G/G | 0.408 | 60 | 147 |  | A | 0.401 | 118 | G | 0.599 | 176 | 294 |

Freq; frequency, Ref allele; 'reference' allele is the base observed in the reference genome sequence at this location,

Population descriptors:
ASW (A): African ancestry in Southwest USA
CEU (C): Utah residents with Northern and Western European ancestry from the CEPH collection
CHB (H): Han Chinese in Beijing, China
CHD (D): Chinese in Metropolitan Denver, Colorado
GIH (G): Gujarati Indians in Houston, Texas
JPT (J): Japanese in Tokyo, Japan
LWK (L): Luhya in Webuye, Kenya
MEX (M): Mexican ancestry in Los Angeles, California
MKK (K): Maasai in Kinyawa, Kenya
TSI (T): Tuscan in Italy
YRI (Y): Yoruban in Ibadan, Nigeri

Table S7 Comparison of previous association studies for AVPR1A polymorphisms in ASD

| Study | Family Sample size | Ethnicity | Significant polymorphism | Key results: Risk allele/haplotype frequency |
| --- | --- | --- | --- | --- |
| Kim et al. (2002) | 115 | Caucasian (94), African (7), and Asian (8)-American  Hispanic (6) | RS3 | Complex (CT)4-TT-(CT)8-(GT)24 marker  (MTDT χ^2^ = 17.05, *P* = 0.048) |
| Wassink et al. (2004) | 65 | Not specified | RS1 | 312, all family, TDT *P* = 0.008 |
|  |  |  | RS3 | 328, language normal subgroup, TDT *P* = 0.01 |
| Yirmiya et al. (2006) | 116 | Not specified | Haplotype AVR-RS1-RS3 | 212-314-325 –most common haplotype (FBAT)  214-310-329 (*P* = 0.01) - lowest *P* value |
| Yang et al. (2010) | 151 | Korean | RS1 | 332 bp allele *P* < 0.001 |
|  |  |  | RS3 | (GATA)_10_ allele  *P* = 0.015 |
| Yang et al. (2010) | 151 | Korean | rs7294536 | A, *P* = 0.002 |
|  |  |  | rs10877969 | A, *P* < 0.001 |
| Tansey et al. (2011) | 177 | Irish | RS1 (short alleles), | RS1, Allele < 302 bp; *P* = 0.036 |
|  |  |  | rs11174815 | A, *P* = 0.008 |
| Kantojärvi  et al. (2015) | 205 | Finnish | RS1 (short alleles) | 306,  *P* = 0.037 |
|  |  |  | Haplotype rs7307997- rs1042615 | G-T lowest *P* = 0.0007, A-T lowest *P* = 0.002 |
|  |  |  | Haplotype  RS3-rs1042615 | 310-T *P* = 0.026 |
